# Effects of Age on Na^+^,K^+^-ATPase Expression in Human and Rodent Skeletal Muscle

**DOI:** 10.3389/fphys.2016.00316

**Published:** 2016-08-02

**Authors:** Victoria L. Wyckelsma, Michael J. McKenna

**Affiliations:** Clinical Exercise Science Program, Institute of Sport Exercise and Active Living, Victoria UniversityMelbourne, VIC, Australia

**Keywords:** age, Na^+^K^+^-pump, single fiber, [^3^H]ouabain

## Abstract

The maintenance of transmembrane Na^+^ and K^+^ concentration gradients and membrane potential is vital for the production of force in skeletal muscle. In aging an inability to maintain ion regulation and membrane potential would have adverse consequences on the capacity for performing repeated muscle contractions, which are critical for everyday activities and functional independence. This short review focusses on the effects of aging on one major and vital component affecting muscle Na^+^ and K^+^ concentrations, membrane potential and excitability in skeletal muscle, the Na^+^,K^+^-ATPase (Na^+^,K^+^-pump, NKA) protein. The review examines the effects of age on NKA in both human and rodent models and highlights a distant lack of research in NKA with aging. In rodents, the muscle NKA measured by [^3^H]ouabain binding site content, declines with advanced age from peak values in early life. In human skeletal muscle, however, there appears to be no age effect on [^3^H]ouabain binding site content in physically active older adults between 55 and 76 years compared to those aged between 18 and 30 years of age. Analysis of the NKA isoforms reveal differential changes with age in fiber-types in both rat and humans. The data show considerable disparities, suggesting different regulation of NKA isoforms between rodents and humans. Finally we review the importance of physical activity on NKA content in older humans. Findings suggest that physical activity levels of an individual may have a greater effect on regulating the NKA content in skeletal muscle rather than aging *per se*, at least up until 80 years of age.

## Implications of impaired skeletal muscle ion regulation in aging

A fundamental factor underpinning skeletal muscle contractile function is the maintenance of membrane excitability, which is heavily dependent on transmembrane sodium (Na^+^), potassium (K^+^), and chloride (Cl^−^) gradients and conductance's via their effects on muscle membrane potential (E_m_) (Hodgkin and Horowicz, 1959). The intramuscular regulation of these ions is important in both the development of and preservation against muscle fatigue (Sejersted and Sjøgaard, 2000; McKenna et al., 2008). Therefore, any disturbances in muscle ion regulation with aging are likely to impact adversely on cellular excitability and the capacity to undertake repeated muscle contractions, thereby affecting the capability to successfully complete simple daily tasks, and thus on quality of life. Preservation of muscle mass and function is critical in older individuals, due their greater risk of falls, and the consequential effects, that include ongoing physical disability, declines in physical and mental health and in social isolation for the individual (Stel et al., 2004). This short review focusses on the effects of aging on one major component of ion regulation in skeletal muscle, the Na^+^,K^+^-ATPase (Na^+^,K^+^-pump, NKA) protein in skeletal muscle, which is vital for the regulation of transmembrane Na^+^ and K^+^ concentration gradients, E_m_ and excitability in skeletal muscle cells (Clausen, 2003b). This review does not cover the acute activation and regulation of the NKA, for this we direct readers to excellent reviews (Clausen, 2003b; Pirkmajer and Chibalin, 2016).

## Na^+^,K^+^-ATPase (NKA) in skeletal muscle

In healthy young adults, the skeletal muscle NKA content is typically around 250–350 pmol.g wet weight^−1^ (Clausen, 2013). Whilst neural tissue contains a higher NKA content per unit mass, skeletal muscle represents the largest pool of NKA in the body due to the very large muscle mass; thus a human weighing 70 kg is estimated to have approximately 8.4 μmol of NKA (Clausen, 2013). The NKA comprises alpha (α) and beta (β) subunits, which together constitute a functional αβ heterodimer (Clausen, 2003b; Green, 2004). The NKA α subunit (~100–112 kDa) contains binding sites for Na^+^, K^+^, and Mg^2+^ ions as well as phosphate and ATP and typically undergoes both phosphorylation and oxidation (Clausen, 2003b; Lingrel et al., 2003; McKenna et al., 2006). The β subunit (~35–55 kDa) is glycosylated and is necessary for the structural maturation of the α subunit, localisation of the NKA heterodimer to the sarcolemma and regulation of NKA activity (Cougnon et al., 2002). Each isoform is encoded by separate genes including four α isoforms (α_1_, α_2_, α_3_, α_4_), and three β isoforms (β_1_, β_2_, β_3_) (Blanco and Mercer, 1998). The expression of these isoforms differs across tissues, which suggests a varying function and regulation of each isoform (McDonough et al., 2002). An additional regulatory (γ) subunit associated with NKA activity, known as the FXYD family of proteins comprising seven isoforms (Geering, 2005); the predominant FXYD isoform in skeletal muscle is FXYD1, or phospholemman (Bibert et al., 2008).

To understand aging effects on NKA in muscle, it is first important to understand distributions of NKA isoforms in specific fiber types. In rat skeletal muscle, based on experiments using isoform-specific antibodies, most studies reveal that the NKA α_1_ isoform has a similar abundance in oxidative and glycolytic muscles (Hundal et al., 1993; Thompson and McDonough, 1996; Ng et al., 2003; Fowles et al., 2004; Zhang et al., 2006; Kristensen and Juel, 2010; Ingwersen et al., 2011). Similarly, the α_2_ is abundant in both oxidative and glycolytic muscles in the rat (Thompson and McDonough, 1996; Fowles et al., 2004; Kristensen and Juel, 2010; Ingwersen et al., 2011). In contrast, rat skeletal muscle exhibits distinct differences in β_1_ and β_2_ expression between fiber types, with an almost exclusive expression of β_1_ in muscles rich in slow twitch fibers, whereas the β_2_ is more abundant in fast twitch fibers (Hundal et al., 1993; Thompson and McDonough, 1996; Fowles et al., 2004; Zhang et al., 2006). The β_3_ isoform in rat muscle was found to be similarly abundant in red and white gastrocnemius muscles (Ng et al., 2003).

In human skeletal muscle, all isoforms except the α_4_ isoform are expressed (Murphy et al., 2004) and recent studies indicate that muscle fiber type NKA expression differs considerably from that in the rat. In human muscle, an initial study found no fiber-type specific differences abundance for the α_1_, β_1_, or FXYD1, but revealed a greater abundance of α_2_ in Type II fibers, with no measures of the other NKA isoforms (Thomassen et al., 2013). Two subsequent studies from our group reported no fiber-type specific abundance in the α_1_, α_2_, and β_1_ isoforms, but a greater abundance of the α_3_ and β_2_ in Type II fibers (Wyckelsma et al., 2015, 2016). The FXYD1 protein was not measured in these latter studies.

The differing abundances of the NKA isoforms between fiber-types in rat muscle and to a lesser extent in human muscle, raises the important issue of understanding what the functional roles of different isoforms are in skeletal muscle. However, our understanding of the specific functions of the NKA isoforms within skeletal muscle is incomplete. Several genetically modified mouse models have been developed to address this, including mice with either one copy of either the α_1_ ($\alpha_{1}^{+/-}$) or α_2_ ($\alpha_{2}^{+/-}$) gene knocked out, leaving the mice with one-half of either the α_1_ or α_2_ isoform compared to wild-type (WT) mice (He et al., 2001), or a skeletal muscle-specific α_2_ gene knock out (skα2^−/−^; Radzyukevich et al., 2012). The extensor digitorum longus (EDL) muscles from $\alpha_{1}^{+/-}$ mice showed reduced muscle force compared with WT mice, while the $\alpha_{2}^{+/-}$ mice had improved force (He et al., 2001). When a fatigue protocol was implemented the $\alpha_{2}^{+/-}$ mouse EDL muscle fatigued at a faster rate than the $\alpha_{1}^{+/-}$ and WT (He et al., 2001). It was suggested the improved force in basal (i.e., non-fatigued) conditions in $\alpha_{2}^{+/-}$ mice could have been explained by altered Ca^2+^ handling in these mice (He et al., 2001). In skα2^−/−^ mice, the resting membrane potential of the EDL was not different to WT mice (Radzyukevich et al., 2012); however, the skα2^−/−^ muscle showed a rapid decline in the maximum twitch and tetanic forces and fatigued earlier during a treadmill running test compared to WT mice (Radzyukevich et al., 2012). These studies now establish, at least in murine skeletal muscle, that the NKA α_1_ plays an important role in Na^+^/K^+^ exchange and E_m_ regulation during basal conditions, presumably in a “house-keeping” role, whilst the α_2_ isoform plays a key role during muscle contractions. The α_3_ is the least abundant of the NKA α isoforms in rat skeletal muscle (Blanco and Mercer, 1998), but its role in skeletal muscle has not yet been established. The α_3_ isoform is the major NKA α isoform expressed in neurones, with NKA α_3_ mutations linked with dystonia-parkinsonism and alternating hemiplegia of childhood (Heinzen et al., 2014). An investigation with α_3_ haplo-insufficiency in mice found that while they could compete physically with WT mice, they were slower in cognitive challenges, such as navigating a water maze (Moseley et al., 2007). Thus, further research is required to determine the functional significance of NKA α_3_ in skeletal muscle.

The overall amount of β isoforms present in a muscle is also important for NKA activity. Muscles expressing NKA α:β isoforms in a ratio of 1:2 had a higher NKA activity than those with a 1:1 ratio, with activity measured by the K^+^-dependent 3-O- methylfluorescein phosphate activity assay (Lavoie et al., 1997). The alpha-beta heterodimer with a β_1_ isoform has a higher affinity to Na^+^ and lower affinity for K^+^ compared to β_2_, independent of the α isoform paired with (Crambert et al., 2000). Whilst the expression of β_1_ and β_2_ isoforms differs between muscle fiber types in the rat, the specific roles of the different β isoforms in skeletal muscle NKA regulation contractile have not yet been defined.

The NKA in muscle is adaptable with disease and physical activity. In humans, declines in skeletal muscle [^3^H]ouabain binding site content have been reported with various diseases including muscular dystrophy (Desnuelle et al., 1982), McArdles disease (Haller et al., 1998), and liver cirrhosis (Aagaard et al., 2002), as well as injury and associated inactivity (Leivseth and Reikerås, 1994; Perry et al., 2015). In contrast, physical training increases muscle [^3^H]ouabain binding site content and is also typically associated with improved exercise performance (McKenna et al., 1996; Clausen, 2013). Given this malleability in muscle NKA and the link with muscular performance, any effects of age on the NKA are of considerable interest.

## Age associated alterations to NKA whole muscle content measured via [^3^H]ouabain binding

The widely accepted method for quantification of the total number of functional NKA in skeletal muscle is through measurement of the [^3^H]ouabain binding site content (Clausen, 2003a). The procedure is performed on small pieces of whole muscle samples (typically between 10 and 20 mg) and is based on the high affinity binding of cardiac glycosides to the α subunit of the NKA, with a stoichiometry of 1:1 (Hansen, 1984). By incubation of muscle samples in tritiated ouabain and counting of β particles via liquid scintillation, it is possible to quantify the NKA content in molar units, typically expressed as NKA in pmol^.^g wet weight^−1^ (Hansen and Clausen, 1988). In human skeletal muscle, the standard [^3^H]ouabain binding assay detects each of the three α isoforms (Wang et al., 2001) and thus this is a measure of NKA content; thus in the article these terms can be read interchangeably. This contrasts rat muscle, where only the α_2_ isoform is detected at the concentration of ouabain used, due to its much higher affinity than the other α isoforms (Hansen, 2001), although this is clearly the most dominantly expressed of the α isoforms (Hansen, 2001). Thus, when referring to rat skeletal muscle the [^3^H]ouabain binding site content is referred rather than the term NKA content which is incorrect.

Few studies have investigated age effects on skeletal muscle [^3^H]ouabain binding site content. From the limited available literature, rodent muscle shows a clear age-dependent effect, although by far the greatest changes are a large upregulation that early occur early in life. In rats, soleus muscle [^3^H]ouabain binding site content increased four-fold from birth up until 1 year of age, which was then followed by a 50–70% decline over the following 2–20 months (Kjeldsen et al., 1984). In rats, soleus muscle exhibited a 58% decrease in [^3^H]ouabain binding site content from rats aged between 28 and 85 days (Kjeldsen et al., 1982). Mice showed less prominent changes in muscle [^3^H]ouabain binding site content than rats. From the first week of life [^3^H]ouabain binding site content increased from around 300 to 800 pmol.g wet weight^−1^ by the 4th week of life, this was followed by a 25% decline which plateaued over the subsequent 4 weeks (Kjeldsen et al., 1984).

There are no lifespan time-course studies investigating chronological changes in human muscle NKA. Several studies utilizing a cross-sectional design have compared muscle NKA content in young healthy participants with a mean age of ~24 years vs. older adults with a mean age of ~68 years (Klitgaard and Clausen, 1989; McKenna et al., 2012; Wyckelsma et al., 2016). The earliest study reported a non-significant (14% lower) difference in NKA content in older compared to the younger adults (Klitgaard and Clausen, 1989). Two recent studies from our group reported no difference in NKA content between young and older adults (McKenna et al., 2012; Wyckelsma et al., 2016). These studies are therefore consistent in finding no significant difference in [^3^H]ouabain binding site content between young and older adults. However, an interesting observation from another recent study was that NKA content was 26% lower in older adults aged between 69 and 81 years compared to those aged from 55 to 68 years (Perry et al., 2013). This suggested that an age associated reduction in muscle NKA content in humans might only be apparent at more advanced ages. To investigate this possible age effect on NKA content further, we analyzed all of the [^3^H]ouabain binding site content data in aging-related research collected in healthy, older participants in our laboratory over the past 4 years (McKenna et al., 2012; Perry et al., 2013; Wyckelsma et al., 2016). The first comparison revealed no difference in NKA content between younger adults aged 18–30 years (*n* = 32) compared to those aged from 55 to 76 years (*n* = 25; 348.3 ± 82.2 vs. 361.8 ± 59.0 pmol.g wet weight^−1^, Young vs. Old, respectively, *p* = 0.53), confirming the above conclusion (Figure 2). Comparison between adults after dividing older adults in subgroups based on decades lived, from 18 to 30 (*n* = 32), 55–59 (*n* = 3), 60–69 (*n* = 11), and 70–76 years of age (*n* = 11), also revealed no differences with age (Figure 2). This suggests that the decline in [^3^H]ouabain binding reported previously in the oldest age category (Perry et al., 2013) may be related to their chronic physical activity levels rather than their age. Older participants in one of the studies reported similar physical activity levels to the healthy young controls, despite the intensities of activities likely differing considerably between the groups (Wyckelsma et al., 2016). This may suggest that preserving some level of physical activity might be the important factor in the maintenance of skeletal muscle NKA with age in humans. Thus, whilst studies in rat and murine muscle suggest that aging is associated with a moderate decline in [^3^H]ouabain binding site content, there is no evidence that this occurs in human skeletal muscle. However, since we have no analyses of muscle [^3^H]ouabain binding in healthy adults greater than 80 years, we cannot exclude the possibility that a decline may occur beyond this age.

## Age associated alterations to NKA isoforms

Despite the [^3^H]ouabain binding site content being the gold standard for measuring NKA content in human muscle, this method (using standard concentration of ouabain) cannot differentiate between the individual α isoforms. Hence researchers are unable to identify which of the three specific α isoforms might have changed with a particular intervention, condition or with aging. Given the possibility of differing roles and fiber-type specificity of the individual isoforms, it is therefore necessary to also investigate the isoform abundances in muscle. This has typically been conducted via western blotting, although one study utilized immunohistochemistry (IHC) to analyze differences in NKA isoforms in aged rat muscle (Zhang et al., 2006). The advantages of IHC are that it allows detection of the cellular localization of specific isoforms and can provide an indication of the fiber-type specificity of a protein. However, disadvantages include the inability to quantify the abundance of protein and the problem of non-specific binding of antibodies.

Few studies have investigated the impacts of aging on NKA isoforms in rat muscle, and the use of different ages of rats and analytical techniques (e.g., western blotting vs. IHC) makes direct comparisons between studies difficult; it is therefore not surprising that findings are inconsistent (Sun et al., 1999; Ng et al., 2003; Zhang et al., 2006). Nonetheless, these studies have identified that aging in rats is associated with skeletal muscle NKA isoform changes that comprise an increased α_1_, either no change or a decline in α_2_, an increase or no change in β_1_, decreased β_2_, and an increases β_3_, as summarized in Table 1. These findings from advanced aged in rodent muscle suggests there may be more NKA α_1_β_1_ heterodimers present in aged skeletal muscle (Sun et al., 1999; Ng et al., 2003). The α_1_β_1_ has a higher Na^+^ affinity in resting muscle (Crambert et al., 2000), and it has been hypothesized that aged rat muscle may have increased K^+^ and Na^+^ fluxes with aging, due to reduced amount of caveolin-3 located in the transverse tubular system of aged mice (Barrientos et al., 2015). This is consistent with the important housekeeping role of the α_1_ isoform in Na^+^/K^+^ exchange. From the above findings and also the reduction of α_2_β_2_ in aged isoforms in rat, it seems reasonable to suggest in aged rats, that the increased abundance of the α_1_β_1_ isoforms may be compensatory for these losses. It was also shown in red gastrocnemius muscle, that α_1_ was less phosphorylated in aged rats (Zhang and Ng, 2007), although the functional implications of this are not yet understood.

**Table 1:** **Effects of age on NKA isoform abundances in skeletal muscle**.

**Ref**	**Species**	**Age (months/years)**	***N***	**Technique (normalization)**	**Muscle Measured**	**NKA Isoform**					
						**α_1_**	**α_2_**	**α_3_**	**β_1_**	**β_2_**	**β_3_**
1	Rat	Young (6 months) Adult (18 months) Old (30 months)	5	Western blot	EDL						
			6		Sol	30 > 6	18 < 6		–		
					RG	30 > 6,18	18, 30 < 6		30 > 6,18	30 < 6	
			7		WG	30 > 6,18	18, 30 < 6		30 > 6,18	30,18 < 6, 30 < 18	
2	Rat	Young (16 months) Old (29 months)	NR	Western blot	EDL	NS	NS		NS		
					Sol						
					RG	↑	NS		NS	↓	↑
					WG	↑	NS		NS	↓	↑
3	Rat	Young (6 months) Old (30 months)	12-15	IHC	RG	↑*	↓*		↑*	↓*	↑*
					WG	↑*	↓*		↑*	↓*	↑*
4	Human	Old 66.8 ± 6.4 Young 23.9 ± 2.2	17 16	Western blot (GAPDH)	VL	**–**	↓ 24%	**–**	**–**	**–**	**–**
5	Human	Old 69.4 ± 3.5	17	Western blot (Calibration Curve)	VL	**–**	**–**	**–**	**–**	**–**	↑ 250%
		Young 25.5 ± 2.8	14		VL Type I fibers	↑ 71%	**–**	**–**	**–**	**–**	↑ 96%
					VL Type II fibers	**–**	**–**	↓ 47%	**–**	↓ 85%	↑ 285%

Reference 1, Sun et al., 1999; 2, Ng et al., 2003; 3, Zhang et al., 2006; 4, McKenna et al., 2012; 5, Wyckelsma et al., 2016.

Age, mean ± SD. Symbols: ↓, denotes decrease; **–,** no change; ↑, increase, data in parentheses denotes % difference between groups. ↑^*^ not quantitative but increased compared to young ↓^*^ not quantitative but decreased compared to young. NR not reported, NS not significant.

Muscles: EDL, Extensor Digitorum Longus; RG, Red Gastrocnemius; WG, White Gastrocnemius; VL, Vastus Lateralis.

Research into the NKA isoform abundances with age in humans is sparse. The initial study by our group investigated NKA isoform abundance in human vastus lateralis muscle homogenates and found a 24% decrease in α_2_ and 23% decrease in β_3_ isoform abundances in older compared to young adults, without change in the other NKA isoforms (McKenna et al., 2012). As there was no difference in [^3^H]ouabain binding site content, it was suggested that the decrease in α_2_ abundance might reflect an increased fiber membrane density due to the smaller fibers in aged muscle (McKenna et al., 2012). A later study revealed a decrease with age in the abundance of several proteins typically used as housekeeping proteins, including glyceraldehyde-3-phosphate dehydrogenase (GAPDH; Vigelsø et al., 2015). The GAPDH protein was used in the above study to normalize NKA western blots (McKenna et al., 2012). We therefore re-examined the effects of age on NKA isoform abundances in muscle homogenates, but normalized against total protein rather than GAPDH and also introduced a calibration curve for normalization (Murphy and Lamb, 2013; Wyckelsma et al., 2016). With this improved method in our subsequent study, we found no difference in the abundance in the α_2_ in aged muscle, which was consistent with the lack of difference in [^3^H]ouabain binding site content. We also observed no differences in other NKA isoforms, apart from a large (~250%) increase in β_3_ isoform abundance (Wyckelsma et al., 2016). This suggests that aging may have quite different effects on the abundance of NKA isoforms in rat and human skeletal muscle.

Since human skeletal muscle comprises a mixed muscle fiber type population, analysis of protein changes in a muscle homogenate might mask changes with aging that occur in different muscle fiber types. We therefore also undertook analysis of NKA isoforms from single fiber segments from biopsies obtained from elderly humans (Wyckelsma et al., 2016). This study firstly showed a lack of fiber-type specificity for any of the six NKA isoforms, with isoforms similarly abundant in Type I and II fibers (Wyckelsma et al., 2016). When isoform abundance was compared between young and older adults in a given fiber type, a number of differences were identified. Compared to young adults, the NKA α_3_ and β_2_ isoforms were both lower by ~47% and ~85%, respectively, in Type II fibers of the older adults, whilst there was a ~71% greater abundance of α_1_ in Type I fibers in aged muscle compared to young. The NKA β_3_ was greater in aged muscle in both Type I (~96%) and II (~285%) fibers, reflective of β_3_ changes seen in the whole muscle homogenate (Wyckelsma et al., 2016).

The greater α_1_ and β_3_ isoforms, and lesser β_2_ isoforms in aged human single fiber segments, showed some similarities with rodent studies (see Table 1). However, no age-associated decreases in the α_2_ or β_1_ have been reported in studies with humans, contrary to some animal studies (Sun et al., 1999). Direct comparison between these studies is difficult due to varying methodological techniques, as well as is the different ages of mice studied. Previous studies utilized mice aged 29 (Ng et al., 2003), and 30 months (Sun et al., 1999; Zhang et al., 2006). Since 13.8 days for a rat is approximately equivalent to 1 year for a human, mice aged 29–30 months would be equivalent to a human aged 75–80 years (Sengupta, 2013). In human physiological research it is quite difficult to recruit older participants of quite advanced age that are in good health, have limited ongoing pharmacological treatments, as well as are willing to undergo invasive procedures, such as muscle sampling. Furthermore, those that do volunteer may already tend to be reasonably active, which might in itself influence expression of NKA α isoforms (Perry et al., 2013).

In human muscle, the increase in the abundance of α_1_ which was commonly seen in rodent has been observed, but no measures of phosphorylation have been investigated. Interestingly all studies with rodents report decreased β_2_ in white and red gastrocnemius muscle (Sun et al., 1999; Ng et al., 2003; Zhang et al., 2006) and also with humans in Type II fibers (Wyckelsma et al., 2016). The role of β_2_ is unknown in skeletal muscle. It is important to explore the role the β_2_ isoform plays in NKA enzymatic activity and excitability, especially in Type II fibers, since Type II fibers undergo a loss of specific force in aged compared to compared to young adults (Lamboley et al., 2015). Whilst FXYD1 phosphorylation has been measured in human muscle (Thomassen et al., 2013, 2016), and increased with exercise in aged rats (Reis et al., 2005), the effects of aging have not yet been investigated in aged humans.

## Chronic regulation of NKA with exercise training in the aged

**Figure 1:**
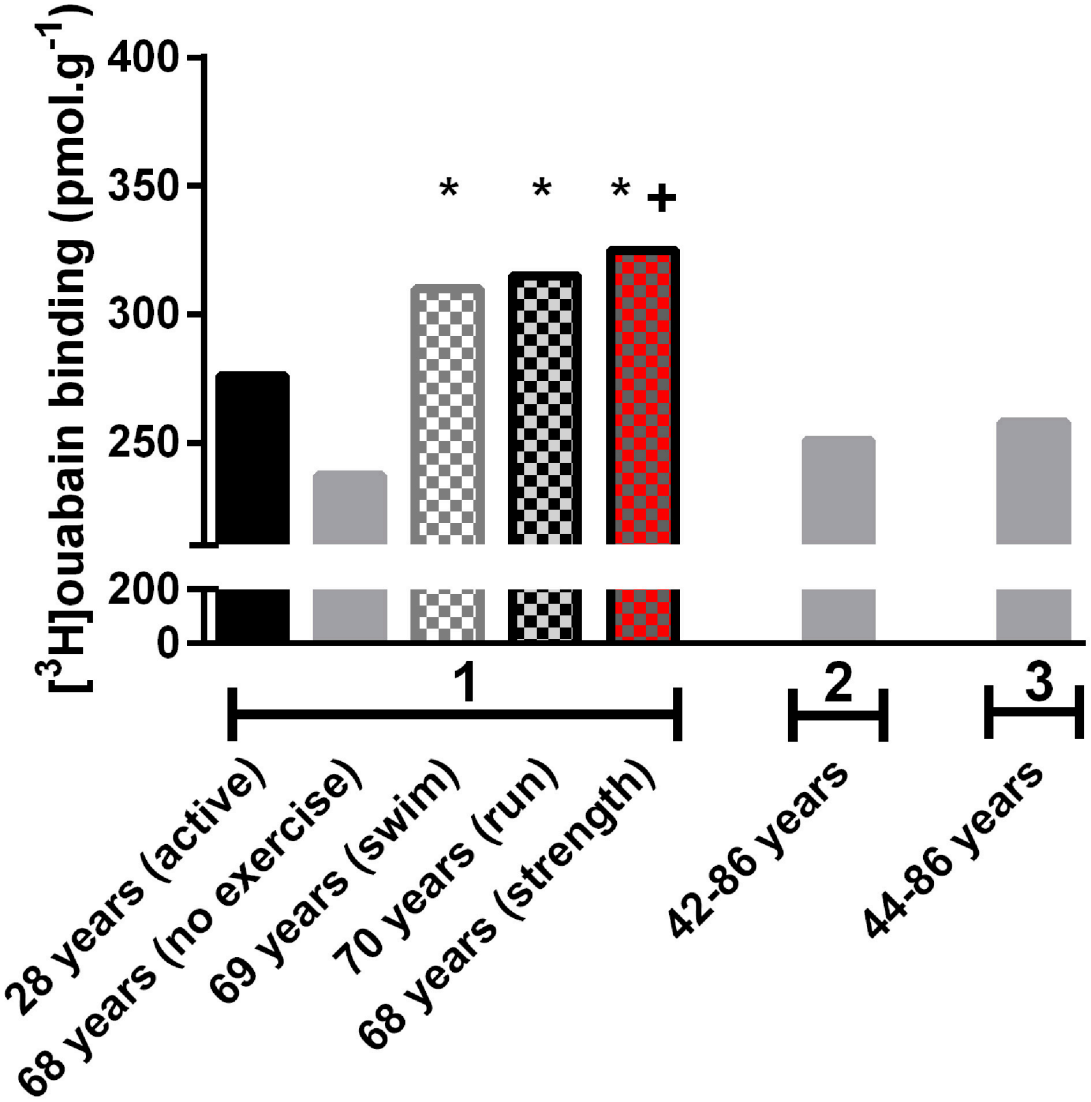
**Human skeletal muscle [^3^H]ouabain binding with aging and physical activity from studies conducted in the Clausen laboratory (Aarhus, Denmark)**. Data from early papers investigating [^3^H]ouabain binding in older adults from different studies measured in the same laboratory to ensure comparisons are made utilizing the same methodology. 1. Indicates data from (Klitgaard and Clausen, 1989), 2. from (Dørup et al., 1988a), and 3. from (Dørup et al., 1988b). Data from 2 to 3 were from healthy control subjects in studies undertaking comparison against clinical populations. These clinical population data have not been included in this figure. *Different to 68 years (no exercise), ^+^ different to young (active), *p* < 0.05.

In young adults, physical activity is known to upregulate the NKA content in skeletal muscle (McKenna et al., 1996; Clausen, 2003b), but little is known about training effects on muscle NKA in the aged. The study by Klitgaard and Clausen (1989), compared [^3^H]ouabain binding site content in healthy but sedentary older and younger adults with three cohorts of active older adults, who had undertaken 12–17 years of regular physical training. The active groups participated in either running, swimming or resistance training and had considerably greater [^3^H]ouabain binding site content than the sedentary older adults (Figure 1). Furthermore, it was found that the resistance trained older adults had a greater [^3^H]ouabain site content compared to untrained young controls (Klitgaard and Clausen, 1989).

**Figure 2:**
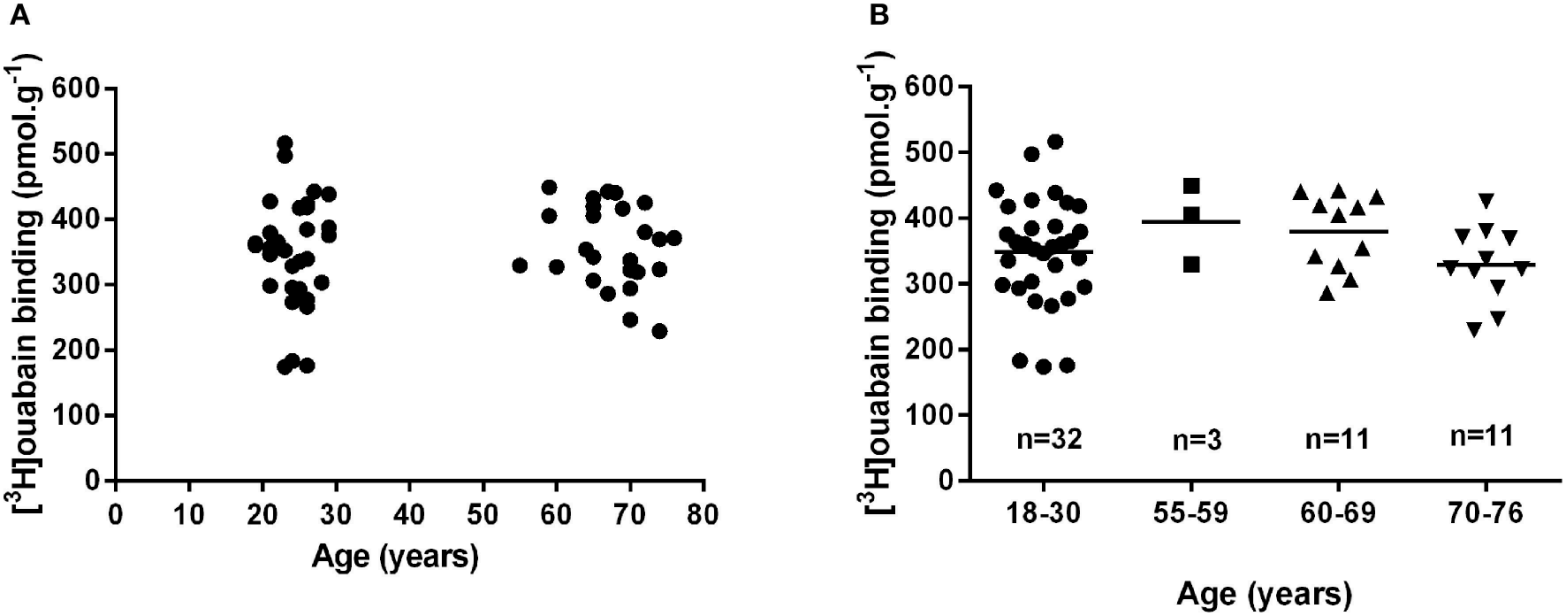
**Muscle [^3^H]ouabain binding does not change with age in human skeletal muscle**. Muscle [^3^H]ouabain binding site content collated from data collected on healthy young and healthy older adults from the McKenna research group between 2012 and 2016. **(A)** Shows the data combined into two discrete age groups and analyzed by unpaired *t*-test (*p* = 0.53). **(B)** Shows all data plotted into relevant decades of life analyzed by one-way ANOVA (*p* = 0.30); the mean of each group is also shown as a horizontal line.

In rats, exercise training further increased the α_1_ isoform, which was already upregulated with age, as measured by western blotting and appeared to reverse the age associated alterations of the NKA β_3_ isoform (Ng et al., 2003) (Table 2). Additional training adaptations included increases in the α_2_ and β_1_ in rat EDL, red and white gastrocnemius muscle (Ng et al., 2003). Another study in aged rats found upregulation of FXYD1 in EDL, red and white gastrocnemius muscle with exercise training (Reis et al., 2005) (Table 2). There were also tendencies for the co-immunoprecipitation of FXYD1 to the α_1_ which tended to decrease with training and the α_2_ tended to increase the co-immunoprecipitation of FXYD1 with training (Reis et al., 2005). However, this study was conducted in only 3 rats, which they suggested may have resulted in a Type II error. The effects of longitudinal training in older adults is yet to be published.

**Table 2:** **Comparison of NKA isoform responses to training in aged rat and human**.

**Ref**	**Species**	**Age**	***N***	**Training**	**Technique**	**Muscle Measured**	**NKA Isoform**						**FXYD1**
							**α_1_**	**α_2_**	**α_3_**	**β_1_**	**β_2_**	**β_3_**	
1	Rat	29 Months	12–15	13–14 weeks motorized treadmill running	Western blot	EDL	↑	↑	↑	**–**	↓	**–**	
						RG	↑	↑	↑	**–**	↓		
						WG		↓	↓	**–**			
2	Rat	29 months	3	13–14 weeks motorized treadmill running	Western blot	EDL							↑
						RG							↑
						WG							↑

1, Ng et al., 2003; 2, Reis et al., 2005. Blank space indicated not measured, ↑ increased ↓ decreased – no change.

Muscles: EDL, Extensor Digitorum Longus; RG, Red Gastrocnemius; WG, White Gastrocnemius; VL, Vastus Lateralis.

## Perspectives

The review highlights the lack of research in aging and NKA regulation in skeletal muscle, with many aspects of NKA regulation still required to be explored. It further demonstrates that changes that might be observed with age in rodent muscles cannot be anticipated to also occur in humans and the reasons for this are unclear. This highlights the importance of conducting studies in human muscle. Such research is particularly important given the vital role of NKA in regulation muscle excitability and function. The use of skeletal muscle knockout mice provide excellent models to determine function of the specific NKA isoforms in skeletal muscle. Future research could consider the effects of aging utilizing these knockout mice to investigate muscle NKA, contractility and function, and including with α_3_, and β_1−3_ isoform knock out models. Until the roles of the NKA isoforms in skeletal muscle are more fully understood, the functional translation of findings in rodent to human muscle physiology can only be speculative. Further research should in addition explore the acute activation of the skeletal muscle NKA in aged muscle and its regulation, as well as the impacts on muscle E_m_, excitability and the link to function during repeated contractions. Finally, any future work with NKA regulation in aged human skeletal muscle should include measures in single fiber segments to ensure that any fiber-type specific responses are not masked.

## Author contributions

VW and MM drafted, edited and approved the final version of the manuscript.

### Conflict of interest statement

The authors declare that the research was conducted in the absence of any commercial or financial relationships that could be construed as a potential conflict of interest.

## References

[B1] Aagaard N. K. Andersen H. Vilstrup H. Clausen T. Jakobsen J. Dørup I. (2002). Muscle strength, Na,K-pumps, magnesium and potassium in patients with alcoholic liver cirrhosis – relation to spironolactone. J. Intern. Med. 252, 56–63. 10.1046/j.1365-2796.2002.01008.x 12074739

[B2] Barrientos G. Llanos P. Hidalgo J. Bolaños P. Caputo C. Riquelme A.. (2015). Cholesterol removal from adult skeletal muscle impairs excitation-contraction coupling and aging reduces caveolin-3 and alters the expression of other triadic proteins. Front. Physiol. 6:105. 10.3389/fphys.2015.00105 25914646 PMC4392612

[B3] Bibert S. Roy S. Schaer D. Horisberger J.-D. Geering K. (2008). Phosphorylation of phospholemman (FXYD1) by protein kinases, A and C modulates distinct Na,K-ATPase isozymes. J. Biol. Chem. 283, 476–486. 10.1074/jbc.M705830200 17991751

[B4] Blanco G. Mercer R. W. (1998). Isozymes of the Na-K-ATPase: heterogeneity in structure, diversity in function. Am. J. Physiol. 275(5 Pt 2), F633–F650. 9815123 10.1152/ajprenal.1998.275.5.F633

[B5] Clausen T. (2003a). Effects of age and exercise training on Na^+^-K^+^ pumps in skeletal muscle. Am. J. Physiol. Regul. Integr. Comp. Physiol. 285, R720–R721. 10.1152/ajpregu.00357.2003 12959916

[B6] Clausen T. (2003b). Na^+^-K^+^ pump regulation and skeletal muscle contractility. Physiol. Rev. 83, 1269–1324. 10.1152/physrev.00011.2003 14506306

[B7] Clausen T. (2013). Quantification of Na^+^,K^+^ pumps and their transport rate in skeletal muscle: functional significance. J. Gen. Physiol. 142, 327–345. 10.1085/jgp.201310980 24081980 PMC3787770

[B8] Cougnon M. Moseley A. Radzyukevich T. Lingrel J. Heiny J. (2002). Na,K-ATPase a- and ß-isoform expression in developing skeletal muscles: a&lt;SUB&gt;2&lt;/SUB&gt; correlates with t-tubule formation. Pflügers Arch. 445, 123–131. 10.1007/s00424-002-0898-6 12397396

[B9] Crambert G. Hasler U. Beggah A. T. Yu C. Modyanov N. N. Horisberger J. D.. (2000). Transport and pharmacological properties of nine different human Na,K-ATPase Isozymes. J. Biol. Chem. 275, 1976–1986. 10.1074/jbc.275.3.1976 10636900

[B10] Desnuelle C. Lombet A. Serratrice G. Lazdunski M. (1982). Sodium channel and sodium pump in normal and pathological muscles from patients with myotonic muscular dystrophy and lower motor neuron impairment. J. Clin. Invest. 69, 358–367. 10.1172/JCI110459 6276440 PMC370985

[B11] Dørup I. Skajaa K. Clausen T. (1988a). A simple and rapid method for the determination of the concentrations of magnesium, sodium, potassium and sodium, potassium pumps in human skeletal muscle. Clin. Sci. 74, 241–248. 10.1042/cs0740241 2449993

[B12] Dørup I. Skajaa K. Clausen T. Kjeldsen K. (1988b). Reduced concentrations of potassium, magnesium, and sodium-potassium pumps in human skeletal muscle during treatment with diuretics. Br. Med. J. 296, 455–458. 10.1136/bmj.296.6620.455 2450616 PMC2545041

[B13] Fowles J. R. Green H. J. Ouyang J. (2004). Na^+^-K^+^-ATPase in rat skeletal muscle: content, isoform, and activity characteristics. J. Appl. Physiol. 96, 316–326. 10.1152/japplphysiol.00745.2002 12882989

[B14] Geering K. (2005). Function of FXYD proteins, regulators of Na, K-ATPase. J. Bioenerg. Biomembr. 37, 387–392. 10.1007/s10863-005-9476-x 16691470

[B15] Green H. J. (2004). Membrane excitability, weakness, and fatigue. Can. J. Appl. Physiol. 29, 291–307. 10.1139/h04-020 15199228

[B16] Haller R. G. Clausen T. Vissing J. (1998). Reduced levels of skeletal muscle Na^+^K^+^-ATPase in McArdle disease. Neurology 50, 37–40. 10.1212/WNL.50.1.37 9443454

[B17] Hansen O. (1984). Interaction of cardiac glycosides with (Na^+^ + K^+^)-activated ATPase. A biochemical link to digitalis-induced inotropy. Pharmacol. Rev. 36, 143–163. 6093155

[B18] Hansen O. (2001). The α1 isoform of Na^+^,K^+^-ATPase in rat soleus and extensor digitorum longus. Acta Physiol. Scand. 173, 335–341. 10.1046/j.1365-201X.2001.00910.x 11736695

[B19] Hansen O. Clausen T. (1988). Quantitative determination of Na^+^-K^+^-ATPase and other sarcolemmal components in muscle cells. Am. J. Physiol. 254, C1–C7. 2447793 10.1152/ajpcell.1988.254.1.C1

[B20] He S. Shelly D. A. Moseley A. E. James P. F. James J. H. Paul R. J.. (2001). The α1- and α2-isoforms of Na-K-ATPase play different roles in skeletal muscle contractility. Am. J. Physiol. Regul. Integr. Comp. Physiol. 281, R917–R925. 11507009 10.1152/ajpregu.2001.281.3.R917

[B21] Heinzen E. L. Arzimanoglou A. Brashear A. Clapcote S. J. Gurrieri F. Goldstein D. B.. (2014). Distinct neurological disorders with ATP1A3 mutations. Lancet Neurol. 13, 503–514. 10.1016/S1474-4422(14)70011-0 24739246 PMC4238309

[B22] Hodgkin A. L. Horowicz P. (1959). The influence of potassium and chloride ions on the membrane potential of single muscle fibres. J. Physiol. 148, 127–160. 10.1113/jphysiol.1959.sp006278 14402240 PMC1363113

[B23] Hundal H. S. Marette A. Ramlal T. Liu Z. Klip A. (1993). Expression of β subunit isoforms of the Na^+^,K^+^-ATPase is muscle type-specific. FEBS Lett. 328, 253–258. 10.1016/0014-5793(93)80938-Q 8394248

[B24] Ingwersen M. Kristensen M. Pilegaard H. Wojtaszewski J. Richter E. Juel C. (2011). Na,K-ATPase activity in mouse muscle is regulated by AMPK and PGC-1α. J. Membr. Biol. 242, 1–10. 10.1007/s00232-011-9365-7 21687978

[B25] Kjeldsen K. Nørgaard A. Clausen T. (1982). Age-dependent changes in the number of [^3^H]ouabain-binding sites in rat soleus muscle. Biochim. Biophys. Acta 686, 253–256. 6282328 10.1016/0005-2736(82)90121-3

[B26] Kjeldsen K. Nøgaard A. Clausen T. (1984). The age-dependent changes in the number of 3H-ouabain binding sites in mammalian skeletal muscle. Pflugers Arch. 402, 100–108. 10.1007/BF00584838 6095173

[B27] Klitgaard H. Clausen T. (1989). Increased total concentration of Na-K pumps in vastus lateralis muscle of old trained human subjects. J. Appl. Physiol. 67, 2491–2494. 2606857 10.1152/jappl.1989.67.6.2491

[B28] Kristensen M. Juel C. (2010). Na^+^,K^+^-ATPase Na^+^ affinity in rat skeletal muscle fiber types. J. Membr. Biol. 234, 35–45. 10.1007/s00232-010-9237-6 20177668

[B29] Lamboley C. R. Wyckelsma V. L. Dutka T. L. McKenna M. J. Murphy R. M. Lamb G. D. (2015). Contractile properties and sarcoplasmic reticulum calcium content in type I and type II skeletal muscle fibres in active aged humans. J. Physiol. 593, 2499–2514. 10.1113/JP270179 25809942 PMC4461411

[B30] Lavoie L. Levenson R. Martin-Vasallo P. Klip A. (1997). The molar ratios of alpha and beta subunits of the Na^+^-K^+^-ATPase differ in distinct subcellular membranes from rat skeletal muscle. Biochemistry 36, 7726–7732. 10.1021/bi970109s 9201913

[B31] Leivseth G. Reikerås O. (1994). Changes in muscle fiber cross-sectional area and concentrations of Na,K-ATPase in deltoid muscle in patients with impingement syndrome of the shoulder. J. Orthop. Sports Phys. Ther. 19, 146–149. 10.2519/jospt.1994.19.3.146 8156065

[B32] Lingrel J. Moseley A. M. Y. Dostanic I. V. A. Cougnon M. He S. James P.. (2003). Functional roles of the α isoforms of the Na,K-ATPase. Ann. N.Y. Acad. Sci. 986, 354–359. 10.1111/j.1749-6632.2003.tb07214.x 12763850

[B33] McDonough A. A. Thompson C. B. Youn J. H. (2002). Skeletal muscle regulates extracellular potassium. Am. J. Physiol. Renal Physiol. 282, F967–F974. 10.1152/ajprenal.00360.2001 11997312

[B34] McKenna M. J. Bangsbo J. Renaud J. M. (2008). Muscle K^+^, Na^+^, and Cl^−^ disturbances and Na^+^-K^+^ pump inactivation: implications for fatigue. J. Appl. Physiol. 104, 288–295. 10.1152/japplphysiol.01037.2007 17962569

[B35] McKenna M. J. Harmer A. R. Fraser S. F. Li J. L. (1996). Effects of training on potassium, calcium and hydrogen ion regulation in skeletal muscle and blood during exercise. Acta Physiol. Scand. 156, 335–346. 10.1046/j.1365-201X.1996.199000.x 8729694

[B36] McKenna M. J. Medved I. Goodman C. A. Brown M. J. Bjorksten A. R. Murphy K. T.. (2006). N-acetylcysteine attenuates the decline in muscle Na^+^,K^+^-pump activity and delays fatigue during prolonged exercise in humans. J. Physiol. 576, 279–288. 10.1113/jphysiol.2006.115352 16840514 PMC1995650

[B37] McKenna M. J. Perry B. D. Serpiello F. R. Caldow M. K. Levinger P. Cameron-Smith D.. (2012). Unchanged [3H]ouabain binding site content but reduced Na^+^-K^+^ pump α2-protein abundance in skeletal muscle in older adults. J. Appl. Physiol. 113, 1505–1511. 10.1152/japplphysiol.01032.2011 22936730

[B38] Moseley A. E. Williams M. T. Schaefer T. L. Bohanan C. S. Neumann J. C. Behbehani M. M.. (2007). Deficiency in Na,K-ATPase alpha isoform genes alters spatial learning, motor activity, and anxiety in mice. J. Neurosci. 27, 616–626. 10.1523/JNEUROSCI.4464-06.2007 17234593 PMC6672804

[B39] Murphy K. T. Snow R. J. Petersen A. C. Murphy R. M. Mollica J. Lee J. S.. (2004). Intense exercise up-regulates Na^+^,K^+^-ATPase isoform mRNA, but not protein expression in human skeletal muscle. J. Physiol. 556, 507–519. 10.1113/jphysiol.2003.054981 14754991 PMC1664937

[B40] Murphy R. M. Lamb G. D. (2013). Important considerations for protein analyses using antibody based techniques: down-sizing Western blotting up-sizes outcomes. J. Physiol. 591, 5823–5831. 10.1113/jphysiol.2013.263251 24127618 PMC3872754

[B41] Ng Y. C. Nagarajan M. Jew K. N. Mace L. C. Moore R. L. (2003). Exercise training differentially modifies age-associated alteration in expression of Na^+^-K^+^-ATPase subunit isoforms in rat skeletal muscles. Am. J. Physiol. Regul. Integr. Comp. Physiol. 285, R733–R740. 10.1152/ajpregu.00266.2003 12805093

[B42] Perry B. D. Levinger P. Morris H. G. Petersen A. C. Garnham A. P. Levinger I.. (2015). The effects of knee injury on skeletal muscle function, Na^+^, K^+^-ATPase content, and isoform abundance. Physiol. Rep. 3:e12294. 10.14814/phy2.12294 25677549 PMC4393202

[B43] Perry B. D. Levinger P. Serpiello F. R. Caldow M. K. Cameron-Smith D. Bartlett J. R.. (2013). The effects of osteoarthritis and age on skeletal muscle strength, Na^+^-K^+^-ATPase content, gene and isoform expression. J. Appl. Physiol. 115, 1443–1449. 10.1152/japplphysiol.00789.2013 24009010

[B44] Pirkmajer S. Chibalin A. V. (2016). Na,K-ATPase regulation in skeletal muscle. Am. J. Physiol. Endocrinol. Metab. 311, E1–E31. 10.1152/ajpendo.00539.2015 27166285

[B45] Radzyukevich T. L. Neumann J. C. Rindler T. N. Oshiro N. Goldhamer D. J. Lingrel J. B.. (2012). Tissue-specific role of the Na,K-ATPase 2 isozyme in skeletal muscle. J. Biol. Chem. 288, 1226–1237. 10.1074/jbc.M112.424663 23192345 PMC3543005

[B46] Reis J. Zhang L. Cala S. Jew K. N. Mace L. C. Chung L.. (2005). Expression of phospholemman and its association with Na^+^-K^+^-ATPase in skeletal muscle: effects of aging and exercise training. J. Appl. Physiol. 99, 1508–1515. 10.1152/japplphysiol.00375.2005 15961612

[B47] Sejersted O. M. Sjøgaard G. (2000). Dynamics and consequences of potassium shifts in skeletal muscle and heart during exercise. Physiol. Rev. 80, 1411–1481. 11015618 10.1152/physrev.2000.80.4.1411

[B48] Sengupta P. (2013). The laboratory rat: relating its age with human's. Int. J. Prev. Med. 4, 624–630. 23930179 PMC3733029

[B49] Stel V. S. Smit J. H. Pluijm S. M. F. Lips P. (2004). Consequences of falling in older men and women and risk factors for health service use and functional decline. Age Ageing 33, 58–65. 10.1093/ageing/afh028 14695865

[B50] Sun X. Nagarajan M. Beesley P. W. Ng Y.-C. (1999). Age-associated differential expression of Na^+^-K^+^-ATPase subunit isoforms in skeletal muscles of F-344/BN rats. J. Appl. Physiol. 87, 1132–1140. 10484587 10.1152/jappl.1999.87.3.1132

[B51] Thomassen M. Gunnarsson T. P. Christensen P. M. Pavlovic D. Shattock M. J. Bangsbo J. (2016). Intensive training and reduced volume increases muscle FXYD1 expression and phosphorylation at rest and during exercise in athletes. Am. J. Physiol. Regul. Intergr. Comp. Physiol. 310, R659–R669. 10.1152/ajpregu.00081.2015 26791827 PMC4867379

[B52] Thomassen M. Murphy R. M. Bangsbo J. (2013). Fibre type-specific change in FXYD1 phosphorylation during acute intense exercise in humans. J. Physiol. 591, 1523–1533. 10.1113/jphysiol.2012.247312 23359667 PMC3607170

[B53] Thompson C. B. McDonough A. A. (1996). Skeletal muscle Na,K-ATPase alpha and beta subunit protein levels respond to hypokalemic challenge with isoform and muscle type specificity. J. Biol. Chem. 271, 32653–32658. 10.1074/jbc.271.51.32653 8955095

[B54] Vigelsø A. Dybboe R. Hansen C. N. Dela F. Helge J. W. Guadalupe Grau A. (2015). GAPDH and β-actin protein decreases with aging, making Stain-Free technology a superior loading control in Western blotting of human skeletal muscle. J. Appl. Physiol. 118, 386–394. 10.1152/japplphysiol.00840.2014 25429098

[B55] Wang J. Velotta J. B. McDonough A. A. Farley R. A. (2001). All human Na^+^-K^+^-ATPase α-subunit isoforms have a similar affinity for cardiac glycosides. Am. J. Physiol.Cell Physiol. 281, C1336–C1343. 11546672 10.1152/ajpcell.2001.281.4.C1336

[B56] Wyckelsma V. L. McKenna M. J. Levinger I. Petersen A. C. Lamboley C. R. Murphy R. M. (2016). Cell specific differences in the protein abundances of GAPDH and Na(^+^),K(^+^)-ATPase in skeletal muscle from aged individuals. Exp. Gerontol. 75, 8–15. 10.1016/j.exger.2015.12.010 26747222

[B57] Wyckelsma V. L. McKenna M. J. Serpiello F. R. Lamboley C. R. Aughey R. J. Stepto N. K.. (2015). Single-fiber expression and fiber-specific adaptability to short-term intense exercise training of Na^+^-K^+^-ATPase alpha- and beta-isoforms in human skeletal muscle. J. Appl. Physiol. 118, 699–706. 10.1152/japplphysiol.00419.2014 25614596

[B58] Zhang L. Ng Y. C. (2007). Fiber specific differential phosphorylation of the α1-subunit of the Na^+^,K^+^-ATPase in rat skeletal muscle: the effect of aging. Mol. Cell. Biochem. 303, 231–237. 10.1007/s11010-007-9479-5 17457517

[B59] Zhang L. Morris K. J. Ng Y.-C. (2006). Fiber type-specific immunostaining of the Na^+^,K^+^-ATPase subunit isoforms in skeletal muscle: age-associated differential changes. Biochim. Biophys. Acta 1762, 783–793. 10.1016/j.bbadis.2006.08.006 16979878 PMC1761903

